# A lack of financial planning predicts increased mortality risk: Evidence from cohort studies in the United Kingdom and United States

**DOI:** 10.1371/journal.pone.0290506

**Published:** 2023-09-27

**Authors:** Joe J. Gladstone, C. Sean Hundtofte

**Affiliations:** 1 Marketing Division, Leeds School of Business, University of Colorado Boulder, Boulder, Colorado, United States of America; 2 Solve Finance, Nyack, New York, United States of America; Sungkyunkwan University School of Social Sciences, REPUBLIC OF KOREA

## Abstract

We investigate whether a lack of planning and future-orientation in financial behavior is associated with a higher mortality risk. Our evidence is based on two nationally representative cohorts of older people living in the United States (*n* = 11,478) and England (*n* = 11,298), where we compared individuals’ self-reported planning horizons on spending and saving with government mortality records. Controlling for demographics, participants with a 1 SD shorter planning horizon had a 9% greater hazard of dying in the English sample (evaluated over 10 years), and a 7% greater hazard in the US sample (over 22 years). These differences in mortality risk could not be explained by variation in respondent’s life expectancy, their financial circumstances or a range of other observable covariates. Similar results are found for self-reported health, with the positive association between longer planning horizons and health strongest for those with fewest financial resources.

## Introduction

Substantial inequality exists in mortality across socio-economic groups. For example, in the U.S., recent estimates of the gap in life expectancy between the richest and poorest populations is as large as 20 years [1, 2]. Proposed mechanisms to explain this relationship have included the psychological consequences of greater economic inequality, or the capacity to spend on healthcare [3]. However, one mechanism which has received little empirical attention is an individual’s future orientation, specifically how far into the future an individual considers the trade-offs of their consumption and savings behavior today. In this research, we test whether differences in forward-looking behavior can help explain variation in health and mortality. To do this, we measure individuals’ planning horizons using two nationally representative surveys of older people living in the United States and England, and compare these survey responses to official government death records in both countries (the National Death Index in the US, and the National Health Service Central Data Registry in the UK). We ask whether people who fail to make far-sighted financial plans are indeed at a greater risk of suffering from adverse health and increased mortality.

Our research is motivated by an important policy challenge; that many households struggle to plan for the future and remain chronically underprepared for the financial burdens of living into older age [4, 5]. Research in psychology and behavioral economics can provide an explanation for this financial unpreparedness: people struggle to plan ahead because they are generally biased towards the present [6–8]. In studies where decisions are made over consuming now or in the future, people display irrational levels of impatience [7]. While under standard theory in Economics, people consider all periods of their life-cycle albeit with different weights (discount factors) to arrive at optimal consumption and savings plans, empirical research demonstrates that households often deviate from optimal solutions and display a lack of “financial sophistication” [9]. Two immediate potential deviations from this standard model of life-cycle planning are inconsistency in time preferences, and to what extent an individual considers future periods, i.e., over what horizons do individuals make plans and consider present/future tradeoffs. In this research, we focus on the latter, with the present study being the first to examine whether a lack of consideration of future periods in financial decision-making–what we refer to as “financial planning”—is associated with a higher mortality risk.

Given that the relationship between planning and mortality is a topic where experiments, whether natural or controlled, are difficult, if not impossible, to perform, we instead rely on observational evidence including government mortality records across two countries. The use of rich panel data allows us to construct and test more complex behavioral hypotheses that follow from causal mechanisms. We hypothesized that those who consider longer time horizons in their financial plans will have lower mortality rates, and be in better health, than those with shorter time horizons (Hypothesis 1). Furthermore, we test whether this relationship is robust to the inclusion of covariates that might confound the association linking planning to mortality. Specifically, differences in subjective life expectancies or financial circumstances could directly explain a higher mortality rate amongst those with shorter horizons for their financial decisions. For example, a short planning horizon may be a rational response to a person’s environment if they believe they will only live for a short time into the future. Similarly, research on poverty suggests those lacking in financial resources focus more on the present at the expense of the future [10]. We therefore introduce a range of covariates to determine the extent to which the relationship of planning to mortality reflects other observable confounding factors. We expect that financial planning will remain a statistically significant predictor of mortality when additional variables representing demographics (age, gender, education and ethnicity), subjective life expectancy (the annual mortality risk implied by the respondent’s stated survival probability), and financial circumstances (overall income, debt and wealth) are taken into consideration.

Our expectation that planning may be an important contributor to health and longevity follows from prior research in consumer psychology, which has demonstrated both that people vary in their propensity to plan into the future with money, and that this variation has important consequences [11]. We suggest that individual differences in financial planning may contribute to explaining subsequent mortality rates because those with a greater propensity to make far-sighted plans with their finances could reduce the mental and physical strains of financial stress. After all, individuals who plan ahead tend to build greater financial resilience over their lifetime [12, 13], and previous research suggests that adverse financial circumstances have a negative impact on health even after controlling for socio-economic status, psychosocial, and lifestyle factors [14–17].

We also wanted to better understand whose health may benefit most from long-term planning. Individuals with greater wealth and income may benefit less from planning ahead with their money because they have an *ex-ante* “financial buffer” to income and expenditure shocks [18, 19]. We therefore tested how the relationship between planning and health varies across the wealth and income distribution of the two populations we study. Our motivation for this was guided by resource substitution theory [20]. The theory proposes that the existence of multiple types of resource can make the attainment of desirable social outcomes, such as improved health, less dependent on the presence of any specific resource [21]. For example, education has been found to be more important to the health of people who are otherwise disadvantaged [21, 22]. Extended to our context, resource substitution theory hypothesizes that planning should improve health and mortality rates to a greater degree for those with low incomes and less wealth, as the returns to planning should be greater for those with less margin for error. Specifically, given financial distress has been found to adversely influence health outcomes and mortality [14, 23], far-sighted financial behavior could decrease the likelihood of experiencing adverse health, through improving one’s financial situation and resilience.

Therefore, we test the hypothesis that there is an interaction between planning and financial resources (income and savings), such that planning has a larger effect on health for financially disadvantaged people compared to those who are more advantaged (Hypothesis 2). We expect that the benefits of planning on health and mortality will be absent for those who have greater financial resources, as these individuals are relatively insulated from experiencing financial hardship.

## Method

### Datasets: ELSA and HRS

We investigate the role of financial planning on mortality risk using the English Longitudinal Study of Ageing (ELSA) and the Health and Retirement Study (HRS). The datasets are large (*n* = 11,298 and *n* = 11,478, respectively) and are designed to be representative samples of the older populations in both countries. The datasets include a wide range of covariates, allowing us to gauge the extent that any relationship between mortality and planning horizon is statistically explained by demographics or other *ex-ante* observables. The studies were designed to be broadly comparable in terms of sampling and content [24, 25].

Mortality data for participants from both samples are sourced from respective government databases—the National Death Index in the US, and the National Health Service Central Data Registry in the UK. These records are then matched to the respondents’ survey answers. We utilize the first wave measures of planning and covariates from both studies to predict mortality outcomes in the following waves. For the HRS, our analysis covers 22 years of data from 1992 to 2014, while for ELSA, we examine a decade of data spanning from 2002 to 2012. As is common with longitudinal studies, attrition can pose a challenge in these samples [26]. However, the precision of death records, which provide the exact date of a respondent’s death regardless of their continued participation in future survey waves, means that attrition is not expected to substantially affect our analyses.

The English Longitudinal Study of Ageing was developed by a team of researchers based at University College London, the Institute of Fiscal Studies, and the National Centre for Social Research. The data are lodged with the UK Data Archive. The HRS (Health and Retirement Study) is sponsored by the National Institute on Aging (grant number NIA U01AG009740) and is conducted by the University of Michigan.

Descriptive statistics for both samples are provided in the S1 and S2 Tables. The data required to replicate our research is posted on the Open Science Framework website: https://osf.io/m2jws/?view_only=3eed2eda6fb24a5d8296e5eb89a4c855.

### Measures

#### Planning horizon (continuous measure)

We construct a measure of planning from a question in both the HRS and ELSA datasets which asks respondents for the time horizon over which they typically make their financial decisions: “*In deciding how much of their (family) income to save or spend*, *people are likely to think about different financial planning periods*. *In planning your (family’s) saving and spending*, *which of the following time periods is most important to you (and your partner)*?*”*

This question has been used previously as a marker of more general time perspective [27] and is correlated with a number of other established markers of time perspective [28]. In HRS, respondents can choose from five possible answers, indicating planning timelines ranging from: ‘over the next few months’; ‘the next year’; ‘the next few years’; ‘the next 5–10 years’; or ‘more than 10 years in the future’. ELSA includes the additional option of ‘the next few weeks’, and if no answer suited respondents, they can indicate they ‘do not plan’ or plan ‘day to day’. In both survey samples, the most common response time-period for financial planning is ‘the next few years.’

Table 1 presents a breakdown of responses from both samples. Given the differences in response options between the two datasets, we use z-standardization (Z = (X—μ) / σ) for the planning measure in the regression models to facilitate comparability. The z-score demonstrates the number of standard deviations a data point is from the mean, facilitating comparison across different planning distributions.

**Table 1:** Distribution of planning horizon categories in HRS and ELSA.

	Does Not Plan / Day to Day	The Next Few Weeks	The Next Few Months	The Next Year	The Next Few Years	The Next 5–10 Years	Longer Than 10 Years
HRS	-	-	2,136 (18.61%)	1,239 (10.79%)	3,794 (33.05%)	3,292 (28.68%)	1,017 (8.86%)
ELSA	1301 (11.52%)	1364 (12.07%)	1430 (12.66%)	1726 (15.28%)	2601 (23.02%)	2110 (18.68%)	766 (6.78%)

*Note*. Due to the variance in planning items across the two datasets, scores are z-standardized in our regression analyses to facilitate comparison.

#### Planning horizon (dichotomous measure)

While our main analyses utilize a continuous measure of planning to capture the variability in individuals’ forward-looking tendencies, we adopt a dichotomous approach for interpreting survival curves. This method categorizes participants into ’short-term planners’ ‐ those planning less than a year into the future, and ’long-term planners’ ‐ those planning for a year or longer. This binary division of planning horizons facilitates a more intuitive understanding of how varying planning durations might correspond to different survival outcomes.

#### Mortality

Survey responses are matched with mortality records from the National Death Index in the US, and the National Health Service Central Data Registry in the UK. These records include information about when respondents die regardless of their participation in subsequent waves of the survey. These matches with the national death indexes are highly successful—over 95% of individuals give permission for their records to be linked and are successfully matched [26]. Information on time of death is also supplemented through exit interviews with family members and other government records. Over the study period, 3531 participants (30.76%) died in the US sample (22 years), and 850 (7.52%) in the UK sample (10 years). Although the records include information on the cause of death, our research analyzes only the timing of all-cause mortality.

#### Self-reported health

In addition to mortality, another significant measure used in our study was self-reported health status. This metric plays a crucial role in our analysis as it provides valuable insights into the participants’ perception of their own health. In both datasets, self-reported health was measured through respondents’ self-assessments. Respondents were asked to rate their general health on a scale. In ELSA, participants reported their health as excellent (*n* = 1512, 13.43%), very good (*n* = 3,230, 28.14%), good (*n* = 3,216, 28.02%), fair (*n* = 1,592, 13.87%), or poor (*n* = 792, 7.03%). In HRS, participants reported their health as excellent (*n* = 2,607, 22.71%), very good (*n* = 3,299, 29.29%), good (*n* = 3,529, 31.34%), fair (*n* = 2,130, 18.91%), or poor (*n* = 833, 7.26%) This subjective measure of health has been widely utilized in health-related research due to its demonstrated correlation with more objective health metrics and its predictive value for mortality [29].

#### Demographics

We control for the demographic information of participants, including age, gender, race and education. Detailed measures of education are not directly comparable across samples, we therefore add an indicator variable of whether a respondent has any university-level education (36.78% in HRS, 22.75% in ELSA). Similarly, as the English sample was predominantly white, we include a binary variable representing anyone with a non-white ethnicity (2.51% in ELSA, 19.91% in HRS).

#### Subjective life expectancy

Subjective life expectancy was assessed in the ELSA interview by the question, “*What are the chances (from 0%-100%) you will live to be age X or more*?” If the participant was age 65 or under, *X* = 75; if age 66–69, *X* = 80; and if age 70–74, *X* = 85. The participants were presented with a show card displaying a scale from 0 to 100 to aid their responses. HRS uses a similar question; however, all respondents are asked the same question for ages 75 (*M* = 69.69, *SD* = 7.02) and 85 (*M* = 37.65, *SD* = 8.81). While this makes comparability of the measure across surveys imperfect, to account for the differences across surveys by age, we calculate the annual mortality risk implied by the respondent’s stated survival probability *p*, assuming a constant mortality risk each year over *N* years until age *X* (*subjective expected mortality rate = (100 –p)/N*). For calculating subjective life expectancy in the HRS, we calculate this using the response to the closest upcoming age horizon.

#### Income, debt and wealth

The survey includes detailed measures of household finances. In HRS our analyses include measures (in USD) of annual income, including all income from pensions and investments (*M* = $48,127.01, *SD* = 50,978.79), total non-mortgage debt (*M* = $3234.66, *SD* = 19898.76), as well as total wealth (*M* = $193,708.8, *SD* = 427,517.6). In ELSA, we similarly include measures (in GBP) of annual total income (*M* = £18,687.78, *SD* = 13,836.68), total non-mortgage debt (*M* = 1493.49, *SD* = 5472.82), and total wealth (*M* = £203,741.90, *SD* = 236,092.70). All financial variables were winsorized to reduce the influence of extreme outliers. To improve interpretation and comparability of results across samples, we *z*-standardize these variables in some analyses.

## Results

**Fig 1:**
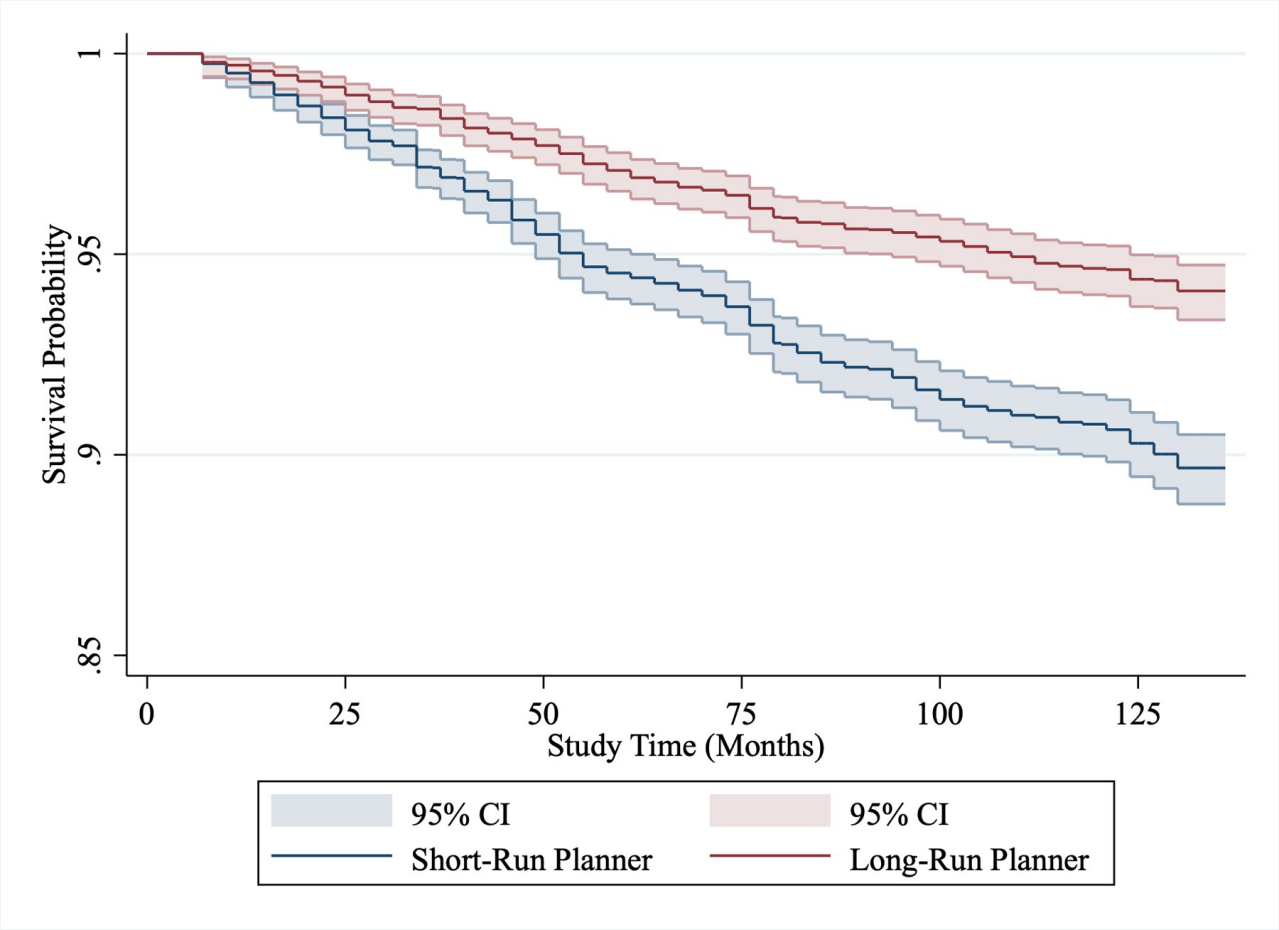
Kaplan-Meier survival curves for long-term vs short-term planners measured in months from baseline (ELSA, UK sample). *Note*: Short-term planners are those who plan a year or less in advance, those planning for more than a year is a long-term planner.

**Fig 2:**
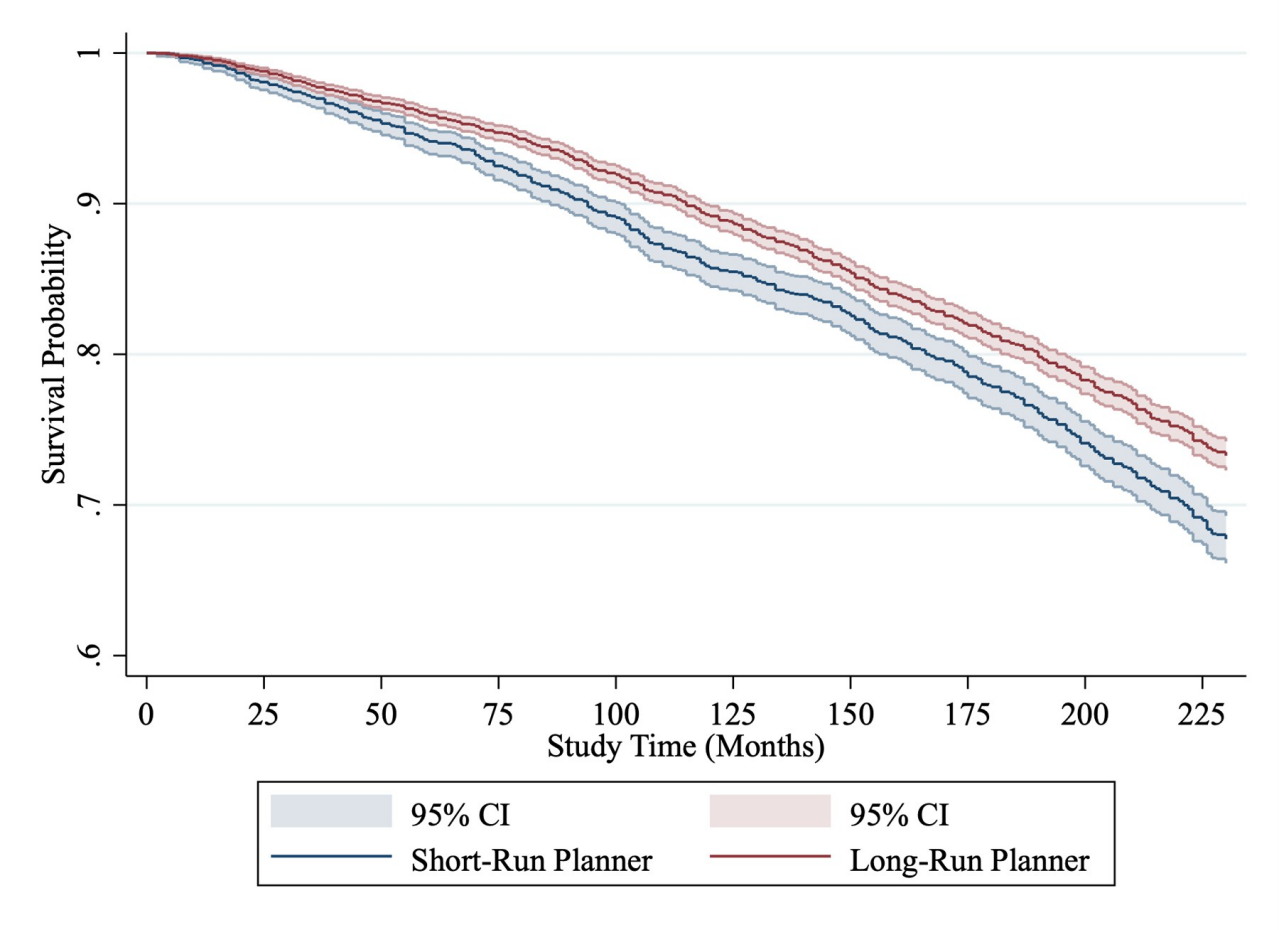
Kaplan-Meier survival curves for long-term vs short-term planners measured in months from baseline (HRS, US sample). *Note*: Short-term planners are those who plan a year or less in advance, those planning for more than a year is a long-term planner.

To begin our examination of whether those who make their financial plans over shorter time horizons have higher mortality rates, we visualize the relationship over time by producing Kaplan-Meier survival curves. In the survival curves, we dichotomize the planning variable to aid interpretation, with those who plan less than a year into the future labelled ‘short-term planners’, while those who plan a year or longer are ‘long-term planners.’ The relationship between planning and mortality is illustrated with Kaplan-Meier survival curves for ELSA and HRS in Figs 1 and 2. The unadjusted relationship between planning and mortality in the ELSA sample is an almost 50% relatively higher risk of death for the short-term planner group over 10 years; with 9.47% of these participants (551 of 5821) dying during the 10-year follow-up period, compared with just 5.46% (299 of 5178) of long-term planners. The relationship between planning and mortality in the HRS sample is smaller, at an almost 20% relatively higher risk of death for the long-term planner group over 22 years; with 35.32% (1192 of 3375) dying in the short-term planner group, while 28.87% (2339 of 8103) dying in the long-term planner group.

To formally test Hypotheses 1, we use a Cox proportional-hazards regression, and report hazard ratios (HRs) with accompanying 95% confidence intervals (CI_95%_). An HR below 1.0 is evidence that the independent variable is associated with a decrease in risk. For example, an HR of 0.75 means that there is a 25% decrease in the continuous hazard rate per unit of measurement in the predictor. Robust estimates of variance are used in all regression models to provide more conservative tests of our hypotheses.

Supporting Hypothesis 1, Models 1 and 5 in Table 2 show that each standard deviation increase in planning is associated with a lower hazard ratio (ELSA, HR = 0.72, CI_95%_ = 0.67–0.77; HRS, HR = 0.88, CI_95%_ = 0.86–0.91), indicating that longer-term planners have a decreased risk of mortality in both the English and US samples.

**Table 2:** Cox proportional hazards models predicting mortality by planning and increasing covariates in ELSA and HRS.

	Hazard Ratios (CI_95%_)							
	ELSA (UK)				HRS (USA)			
Model	(1)	(2)	(3)	(4)	(5)	(6)	(7)	(8)
	Baseline	+Demographics	+SLE	+Finances	Baseline	+Demographics	+SLE	+Finances
Planning Horizon (SD)	0.72***	0.91*	0.91*	0.92*	0.88***	0.93***	0.93***	0.96*
	(0.67 ‐ 0.77)	(0.84 ‐ 0.98)	(0.84 ‐ 0.98)	(0.85 ‐ 0.99)	(0.86 ‐ 0.91)	(0.90 ‐ 0.96)	(0.90 ‐ 0.96)	(0.93 ‐ 0.99)
Age	-	1.08***	1.09***	1.09***	-	1.08***	1.08***	1.08***
		(1.08 ‐ 1.09)	(1.08 ‐ 1.10)	(1.08 ‐ 1.10)		(1.08 ‐ 1.09)	(1.07 ‐ 1.09)	(1.07 ‐ 1.09)
Male	-	0.58***	0.58***	0.57***	-	0.63***	0.63***	0.61***
		(0.50 ‐ 0.66)	(0.50 ‐ 0.66)	(0.50 ‐ 0.65)		(0.59 ‐ 0.68)	(0.59 ‐ 0.68)	(0.57 ‐ 0.66)
Education	-	0.87	0.88	0.92	-	0.68***	0.68***	0.80***
		(0.72 ‐ 1.05)	(0.73 ‐ 1.06)	(0.76 ‐ 1.12)		(0.63 ‐ 0.73)	(0.64 ‐ 0.74)	(0.74 ‐ 0.87)
Non-White	-	0.96	0.97	0.94	-	1.32***	1.32***	1.22***
		(0.56 ‐ 1.62)	(0.57 ‐ 1.64)	(0.56 ‐ 1.60)		(1.22 ‐ 1.43)	(1.22 ‐ 1.43)	(1.13 ‐ 1.33)
Subjective Mortality Risk	-	-	0.99**	0.99**	-	-	1.01^†^	1.01^†^
			(0.98 ‐ 1.00)	(0.99 ‐ 1.00)			(1.00 ‐ 1.03)	(1.00 ‐ 1.03)
Income (SD)	-	-	-	0.99	-	-	-	0.77***
				(0.89 ‐ 1.09)				(0.71 ‐ 0.83)
Debt (SD)	-	-	-	0.92	-	-	-	1.02
				(0.80 ‐ 1.04)				(0.99 ‐ 1.05)
Wealth (SD)	-	-	-	0.90*	-	-	-	0.87**
				(0.81 ‐ 1.00)				(0.81 ‐ 0.95)
AIC	15,620.64	14,897.32	14,888.2	14,886.15	64,755.55	63,570.36	63,564.73	63,411.71
N	11,298				11,478			

*Note*:^†^ *p* < 0.1

^*^ *p* < 0.05

^**^ *p* < 0.01

^***^ *p* < 0.001. Hazard Ratios reported, with 95% robust confidence intervals in parentheses. Planning horizon and financial variables in standard deviation (SD) units.

We next introduce a range of covariates to determine the extent to which the relationship of planning to mortality reflects other observable confounding factors (Models 2–4 and 6–8). Consistent with Hypothesis 1, we find that financial planning remains a statistically significant predictor of mortality when additional variables representing demographics (age, gender, education and ethnicity), subjective mortality risk (the annual mortality risk implied by the respondent’s stated survival probability), and financial circumstances (income from wages, pensions and investments, total debt and total wealth) are taken into consideration.

In Models 4 and 8, which account for all the covariates, we observe an attenuation of the association between planning and mortality, resulting in hazard ratios of 0.92 in ELSA and 0.96 in HRS. While this attenuation effect is evident in both datasets, it is important to consider the variations in the categories of the planning variable, as illustrated in Table 1, as well as the differences in the time periods covered by each dataset. These disparities pose challenges when directly comparing the z-scores between the two samples, as the variations in category distributions may introduce response bias, and different time trends in mortality could confound the effect of planning. Therefore, caution should be exercised when interpreting and comparing the z-scores between HRS and ELSA.

### Resource substitution

As an additional test of Hypotheses 1, we also examined the relationship between planning and self-reported health. We repeat our analyses using OLS regression models predicting self-reported health. As reported in Table 3, we find that each standard deviation increase in planning is associated with improved self-reported health in both ELSA (Model 1, *b* = .14, *t*(11,286) = 12.73, *p* < .001, CI_95%_ = 0.12, 0.16) and HRS (Model 4, *b* = .10, *t*(11,468) = 9.21, *p* < .001, CI_95%_ = 0.08, 0.12).

**Table 3:** OLS regression models predicting self-reported health by planning and covariates in ELSA and HRS, including interactions between planning and financial resources (income and wealth).

	B					
	(CI_95%_)					
	ELSA (UK)			HRS (USA)		
Model	(1)	(2)	(3)	(4)	(5)	(6)
Planning Horizon (SD)	0.14***	0.14***	0.13***	0.10***	0.09***	0.09***
	(0.12 ‐ 0.16)	(0.11 ‐ 0.16)	(0.11 ‐ 0.16)	(0.08 ‐ 0.12)	(0.07 ‐ 0.11)	(0.07 ‐ 0.11)
Age	-0.01***	-0.01***	-0.01***	-0.01***	-0.01***	-0.01***
	(-0.01 - -0.00)	(-0.01 - -0.00)	(-0.01 - -0.00)	(-0.01 - -0.01)	(-0.01 - -0.01)	(-0.01 - -0.01)
Male	0.09***	0.09***	0.09***	0.03	0.03	0.03
	(0.05 ‐ 0.13)	(0.05 ‐ 0.13)	(0.05 ‐ 0.13)	(-0.02 ‐ 0.07)	(-0.02 ‐ 0.07)	(-0.02 ‐ 0.07)
Education	0.16***	0.16***	0.17***	0.36***	0.35***	0.35***
	(0.11 ‐ 0.21)	(0.11 ‐ 0.22)	(0.12 ‐ 0.22)	(0.31 ‐ 0.40)	(0.31 ‐ 0.40)	(0.31 ‐ 0.40)
Non-White	-0.40***	-0.40***	-0.40***	-0.33***	-0.33***	-0.33***
	(-0.53 - -0.28)	(-0.52 - -0.27)	(-0.52 - -0.27)	(-0.38 - -0.28)	(-0.38 - -0.28)	(-0.38 - -0.28)
Subjective Mortality Risk	-0.01***	-0.01***	-0.01***	-0.09***	-0.09***	-0.09***
	(-0.01 - -0.01)	(-0.01 - -0.01)	(-0.01 - -0.01)	(-0.10 - -0.08)	(-0.10 - -0.08)	(-0.10 - -0.08)
Income (SD)	0.08***	0.10***	0.09***	0.22***	0.24***	0.22***
	(0.06 ‐ 0.11)	(0.07 ‐ 0.12)	(0.06 ‐ 0.11)	(0.20 ‐ 0.25)	(0.21 ‐ 0.26)	(0.20 ‐ 0.25)
Debt (SD)	0.03**	0.03*	0.02*	-0.04***	-0.05***	-0.04***
	(0.01 ‐ 0.05)	(0.01 ‐ 0.05)	(0.00 ‐ 0.04)	(-0.06 - -0.02)	(-0.07 - -0.03)	(-0.06 - -0.02)
Wealth (SD)	0.15***	0.16***	0.18***	0.06***	0.06***	0.08***
	(0.13 ‐ 0.18)	(0.13 ‐ 0.18)	(0.16 ‐ 0.21)	(0.04 ‐ 0.08)	(0.04 ‐ 0.09)	(0.06 ‐ 0.11)
Planning X Income	-	-0.03**	-	-	-0.06***	-
		(-0.06 - -0.01)			(-0.09 - -0.04)	
Planning X Wealth	-	-	-0.07***	-	-	-0.06***
			(-0.09 - -0.05)			(-0.08 - -0.04)
AIC	33,266.54	33,258.4	33,226.58	34,430.66	34,393.95	34,399.62
N	11,298			11,478		

*Note*: ^†^ *p* < 0.1

^*^ *p* < 0.05

^**^ *p* < 0.01

^***^ *p* < 0.001. Hazard Ratios reported, with 95% robust confidence intervals in parentheses. Planning horizon and financial variables reported in standard deviation (SD) units.

We next test whether the effect of planning on health varies based on the financial circumstances of the participants (Hypothesis 2). We find that the positive relationship between planning and health is weaker for those who are insulated from experiencing financial hardship because of their wealth or income, as indicated by significant interaction effects between planning and income (ELSA: Model 2, *b* = –.03, *t*(11,285) = -3.18, *p* = .001, CI_95%_ = -0.06, -0.01; HRS: Model 5, *b* = –.06, *t*(11,467) = -6.22, *p* < .001, CI_95%_ = -0.09, -0.04) and wealth (ELSA: Model 3, *b* = –.07, *t*(11,285) = -6.48, *p* < .001, CI_95%_ = -0.09, -0.05; HRS: Model 6, *b* = –.06, *t*(11,467) = -5.75, *p* < .001, CI_95%_ = -0.08, -0.04).

**Fig 3:**
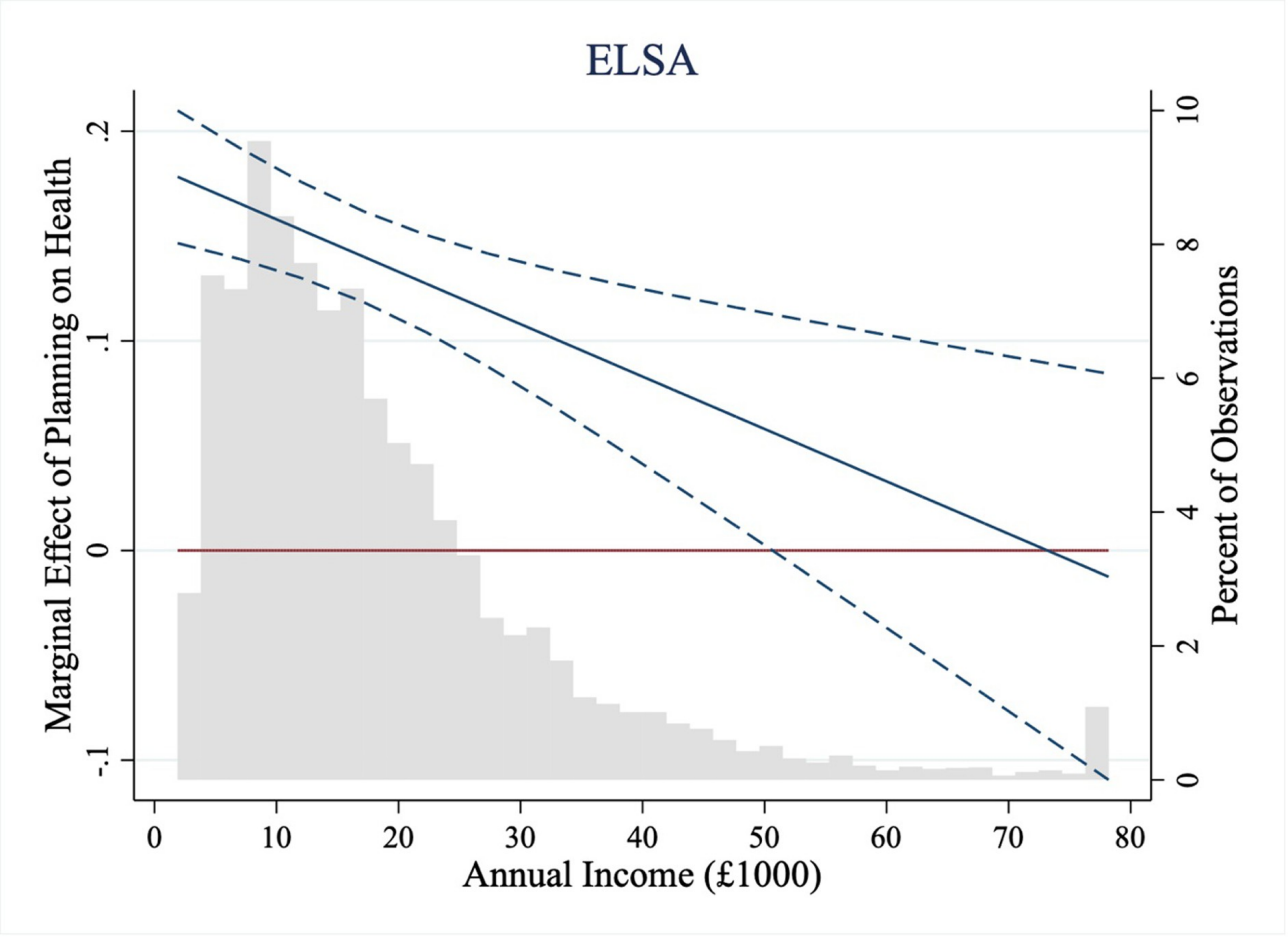
Marginal effect of planning on self-reported health across income in ELSA.

Below a threshold of annual income (approximately £50,000 in ELSA and $80,000 in HRS), and overall wealth (approximately £550,000 in ELSA and $450,000 in HRS) increased planning was significantly positively associated with greater health. We plot the marginal effect of a 1 standard deviation increase in planning on health across the range of income present in the ELSA dataset in Fig 3. The pattern of results is consistent with Hypothesis 2, that planning benefits health for financially disadvantaged people more than the advantaged, because those with greater wealth and income have a financial buffer to income or expenditure shocks [18, 19], insulating them from experiencing financial hardship. These results are consistent with the idea that planning ahead represents an important resource for those with few financial resources, possibly as they do not have the buffer to cope with shocks.

## Discussion

Our results provide evidence that one aspect of forward-looking behavior—a person’s financial planning horizon—is associated with mortality risk, that this difference is of meaningful magnitude, and that the difference in risk cannot be immediately explained by variation in individuals’ (subjective) life expectancy or a wide range of other observables. Our results replicate across two different populations in two different countries, suggesting the results may reflect generalizable differences, rather than a specific culture or healthcare setting. Across the two samples, the pattern in attenuation of mortality outcomes is similar as additional variables are taken into consideration. While the precise differences between the two samples could be for a number of reasons: difference in survey methods; the relative correlations and importance of other factors; or the longer term of the survey in the US, the relative consistency between the two suggests consumer’s spending on healthcare is not a primary mechanism in explaining the relationship between planning and mortality, given the socialized nature of health care in the UK.

An important contribution of the research was to show that the magnitude of the effect of planning on health is moderated by financial resources (income and wealth). Our evidence that wealth moderates the effect suggests that financial distress could be driving part of the relationship we document. Such an interpretation would be in line with previous research suggesting that adverse financial circumstances have a negative impact on health even after controlling for socio-economic status, psychosocial, and lifestyle factors [14–17]. While many complex associations likely shape the relationship between planning and mortality, financial disadvantage may lead people to depend more on the ability to plan, consistent with a resource substitution theory account (Ross and Mirowsky, 2003).

While our results suggest that longer-term financial planning is related to a decreased mortality risk, the results can provide only limited evidence for the mechanism which may underlie this relationship. Further research on this mechanism is important, as understanding the channel through which financial planning predicts mortality might help in formulating effective policy interventions to reduce disparities in health. One mechanism through which planning ahead could improve mortality and health outcomes is through individual consumer’s spending on healthcare, such as long-term planners having a greater demand for preventative treatments designed to reduce future morbidity [3]. For example, increased screening can detect cancer at a stage when it can be effectively treated, improving long-term survival [30, 31] and voluntary screening is more likely to be taken-up by those with a more conscientious personality [32]. Our research compares two nationally representative cohorts of older people living in the United States and England, countries with similar economic and health profiles but with markedly different healthcare systems [33]. These systems can be thought to represent two extremes that exist internationally, the former having the largest private sector system, and the latter with one of the largest public sector systems. All English residents are automatically entitled to free public health care through the National Health Service, including hospital, physician, and mental health care, with the National Health Service budget funded primarily through general taxation. Therefore, if planning ahead could improve mortality and health outcomes through individual consumers’ capacity to spend on healthcare, then we would only expect to see this association in the US context, and certainly not to find it after controlling for wealth.

We note considerable disparities in mortality rates across the two samples, with the HRS dataset exhibiting a higher death rate over the same duration, despite its relatively younger average age. This finding is in line with previous research findings. One possible explanation for this variation could stem from the disparate health conditions of the ELSA and HRS participants. An earlier study suggested that the ELSA cohort was comparatively healthier than the HRS cohort, with fewer instances of severe disability or chronic diseases. As a result, the ELSA group showcased a lower 8-year mortality rate of 14%, contrasting with the HRS development and validation cohorts that reported mortality rates of 23% and 25% respectively [34]. Demographic composition may also factor into this discrepancy, as the HRS encompasses a larger percentage of minority groups, such as African-Americans and Hispanics, who are generally associated with higher mortality rates compared to whites [35]. Lastly, differences in healthcare systems and lifestyle practices between the United States and the United Kingdom might contribute to the observed health outcome variations, including mortality rates, between the two nations [36].

A clear limitation of our findings is that we cannot establish a causal relationship given the datasets and methodology we employ. While the variety and detail of variables used allows us to control for a large number of potential confounding and mediating factors, some factors will not have been considered. Nevertheless, the relationship between planning and mortality is a topic where experiments, whether natural or controlled, are difficult, if not impossible, to perform. Despite these limitations, there are various avenues for future research to build upon and enhance our findings.

One promising direction for future investigations involves exploring the associations between planning and specific causes of death [37]. Both the US National Death Index and the UK National Health Service Central Data Registry provide data on the cause of death, presenting an opportunity to assess whether planning is linked to stress-related mortality. This avenue of research could shed light on the mechanisms through which planning affects mortality, such as healthcare expenditure, preventative behaviors, or medical adherence.

Another direction for future research is to use datasets from more diverse countries to examine the cross-cultural variation in the association between planning and mortality. This could assess the consistency and applicability of our findings, and investigate how cultural factors, such as individualism or collectivism, affect the role of planning in influencing mortality outcomes. For instance, cultural values, social norms, and institutional factors could shape individuals’ long-term planning and their health risks. Comparing these associations across different countries would reveal the cultural specificity of the planning-mortality relationship.

Third, future research could employ alternative or complementary measures of planning that capture different aspects of this construct. For example, instead of relying on self-reported survey items, researchers could use direct observations of individuals’ financial behaviors, such as the time horizon over which they make investments. Alternatively, researchers could use psychometric measures of planning that assess individuals’ cognitive abilities and preferences for planning over different time horizons [11]. These measures could provide more objective and nuanced indicators of planning that could enhance the validity and reliability of the analysis.

In conclusion, increasing longevity, declining birth rates, and government policy changes mean the welfare of an increasingly large proportion of society will depend on the ability of individuals to plan adequately for their financial futures. The evidence we have found in the U.K. and the U.S. of a connection between financial planning and health outcomes highlight the need to develop a more comprehensive understanding of the mechanisms underlying how people’s planning horizons, economic circumstances and longevity are inter-related. The results suggest that financial planning and mitigating financial distress are potential targets for policy interventions seeking to reduce disparities in health outcomes amongst an aging population.

## Supporting information

S1 Table HRS descriptive statistics. (DOCX)

S2 Table ELSA descriptive statistics. (DOCX)

## References

[1] Marmot M. Social determinants of health inequalities. The Lancet. 2005;365: 1099–1104. doi: 10.1016/S0140-6736(05)71146-6 15781105

[2] Chetty R, Stepner M, Abraham S, Lin S, Scuderi B, Turner N, et al. The Association Between Income and Life Expectancy in the United States, 2001–2014. JAMA. 2016;315: 1750. doi: 10.1001/jama.2016.4226 27063997 PMC4866586

[3] Cutler D, Deaton A, Lleras-Muney A. The Determinants of Mortality. Journal of Economic Perspectives. 2006;20: 97–120. doi: 10.1257/jep.20.3.97

[4] Laibson D. Golden eggs and hyperbolic discounting. Quarterly Journal of Economics. 1997;112: 443–477. doi: 10.1162/003355397555253

[5] Mitchell OS, Moore JF. Can Americans Afford to Retire? New Evidence on Retirement Saving Adequacy. ©The Journal of Risk and Insurance. 1998;65.

[6] O’Donoghue T, Rabin M. Doing it now or later. American Economic Review. 1999;89: 103–124. doi: 10.1257/aer.89.1.103

[7] Frederick S, Loewenstein G, O’Donoghue T. Time Discounting and Preference: A Critical Review. Journal of Economic Literature. 2002. pp. 351–401. doi: 10.1126/science.151.3712.867-a 17746758

[8] Malkoc S a, Zauberman G. Deferring Versus Expediting Consumption: The Effect of Outcome Concreteness on Sensitivity to Time Horizon. Journal of Marketing Research. 2006;43: 618–627. doi: 10.1509/jmkr.43.4.618

[9] Hastings JS, Madrian BC, Skimmyhorn WL. Financial literacy, financial education, and economic outcomes. Annual Review of Economics. 2013;5: 347–373. doi: 10.1146/annurev-economics-082312-125807 23991248 PMC3753821

[10] Mullainathan S, Shafir E. Scarcity: Why having too little means so much. New York, NY, US: Times Books/Henry Holt and Co; 2013.

[11] Lynch JG, Netemeyer RG, Spiller SA, Zammit A. A Generalizable Scale of Propensity to Plan: The Long and the Short of Planning for Time and for Money. Journal of Consumer Research. 2010;37: 108–128. doi: 10.1086/649907

[12] Ameriks J, Caplin A, Leahy J. Wealth Accumulation and the Propensity to Plan. The Quarterly Journal of Economics. 2003;118: 1007–1047. doi: 10.1162/00335530360698487

[13] Lusardi A, Mitchell OS. Baby Boomer retirement security: The roles of planning, financial literacy, and housing wealth. Journal of Monetary Economics. 2007;54: 205–224. doi: 10.1016/j.jmoneco.2006.12.001

[14] Molarius A, Berglund K, Eriksson C, Lambe M, Nordström E, Eriksson HG, et al. Socioeconomic conditions, lifestyle factors, and self-rated health among men and women in Sweden. European journal of public health. 2007;17: 125–33. doi: 10.1093/eurpub/ckl070 16751631

[15] Adamson JA, Ebrahim S, Hunt K. The psychosocial versus material hypothesis to explain observed inequality in disability among older adults: data from the West of Scotland Twenty-07 Study. Journal of epidemiology and community health. 2006;60: 974–80. doi: 10.1136/jech.2005.044768 17053287 PMC2465487

[16] Kahn JR, Pearlin LI. Financial strain over the life course and health among older adults. Journal of health and social behavior. 2006;47: 17–31. doi: 10.1177/002214650604700102 16583773

[17] Tucker-Seeley RD, Li Y, Subramanian S V, Sorensen G. Financial hardship and mortality among older adults using the 1996–2004 Health and Retirement Study. Annals of epidemiology. 2009;19: 850–7. doi: 10.1016/j.annepidem.2009.08.003 19944348 PMC2835519

[18] Carroll CD, Samwick AA. The nature of precautionary wealth. Journal of Monetary Economics. 1997;40: 41–71.

[19] Palumbo MG. Uncertain Medical Expenses and Precautionary Saving Near the End of the Life Cycle. Review of Economic Studies. 1999;66: 395–421.

[20] Mirowsky J. Education, Social Status, and Health. 2017. Available: https://www.taylorfrancis.com/books/e/9781351328081

[21] Ross CE, Mirowsky J. Sex differences in the effect of education on depression: Resource multiplication or resource substitution? Social Science & Medicine. 2006;63: 1400–1413. doi: 10.1016/j.socscimed.2006.03.013 16644077

[22] Ross CE, Mirowsky J. Gender and the Health Benefits of Education. The Sociological Quarterly. 2010;51: 1–19. doi: 10.1111/j.1533-8525.2009.01164.x 24288417 PMC3840544

[23] Dobbie W, Song J. Debt relief and debtor outcomes: Measuring the effects of consumer bankruptcy protection. American Economic Review. 2015;105: 1272–1311.

[24] Juster FT, Suzman R. An overview of the Health and Retirement Study. Journal of Human Resources. 1995;30: S7–S56. doi: 10.2307/146277

[25] Steptoe A, Breeze E, Banks J, Nazroo J. Cohort profile: The English Longitudinal Study of Ageing. International Journal of Epidemiology. 2013;42: 1640–1648. doi: 10.1093/ije/dys168 23143611 PMC3900867

[26] Attrition and health in ageing studies: evidence from ELSA and HRS. LLCS. 2011;2. doi: 10.14301/llcs.v2i2.115 24376472 PMC3872999

[27] Picone G, Sloan F, Taylor D. Effects of risk and time preference and expected longevity on demand for medical tests. Journal of Risk and Uncertainty. 2004. doi: 10.1023/B:RISK.0000009435.11390.23

[28] Adams J, Nettle D. Time perspective, personality and smoking, body mass, and physical activity: An empirical study. British Journal of Health Psychology. 2009;14: 83–105. doi: 10.1348/135910708X299664 18435866

[29] McGee DL, Liao Y, Cao G, Cooper RS. Self-reported Health Status and Mortality in a Multiethnic US Cohort. American Journal of Epidemiology. 1999;149: 41–46. doi: 10.1093/oxfordjournals.aje.a009725 9883792

[30] Ahlquist DA. Universal cancer screening: revolutionary, rational, and realizable. npj Precision Oncology. 2018;2. doi: 10.1038/s41698-018-0066-x 30393772 PMC6206005

[31] Lennon AM, Buchanan AH, Kinde I, Warren A, Honushefsky A, Cohain AT, et al. Feasibility of blood testing combined with PET-CT to screen for cancer and guide intervention. Science. 2020;369: eabb9601. doi: 10.1126/science.abb9601 32345712 PMC7509949

[32] Hajek A, Kretzler B, König H-H. Personality and the use of cancer screenings. A systematic review. Luciano M, editor. PLoS ONE. 2020;15: e0244655. doi: 10.1371/journal.pone.0244655 33370379 PMC7769487

[33] Johnson JA, Luo N, Shaw JW, Kind P, Coons SJ. Valuations of EQ-5D Health States: Are the United States and United Kingdom Different? Medical Care. 2005;43: 221–228. doi: 10.1097/00005650-200503000-00004 15725978

[34] Lee SJ, Boscardin WJ, Kirby KA, Covinsky KE. Individualizing Life Expectancy Estimates for Older Adults Using the Gompertz Law of Human Mortality. Berthold HK, editor. PLoS ONE. 2014;9: e108540. doi: 10.1371/journal.pone.0108540 25265291 PMC4180452

[35] Bond Huie SA, Krueger PM, Rogers RG, Hummer RA. Wealth, Race, and Mortality ^*^: Wealth, Race, and Mortality. Social Science Quarterly. 2003;84: 667–684. doi: 10.1111/1540-6237.8403011

[36] Nolte E, McKee CM. In Amenable Mortality—Deaths Avoidable Through Health Care—Progress In The US Lags That Of Three European Countries. Health Affairs. 2012;31: 2114–2122. doi: 10.1377/hlthaff.2011.0851 22933419

[37] Cohen S, Janicki-Deverts D, Miller GE, BS M, SW P, C H, et al. Psychological Stress and Disease. JAMA. 2007;298: 1685. doi: 10.1001/jama.298.14.1685 17925521

