# Supplementary material for: A lack of financial planning predicts increased mortality risk: Evidence from cohort studies in the United Kingdom and United States
Source: PLoS One. 2023 Sep 27;18(9):e0290506. doi: 10.1371/journal.pone.0290506 (PMC10529586; doi:10.1371/journal.pone.0290506)
Supplement: S1 Table — (DOCX) [file pone.0290506.s001.docx]

**S1 Table. HRS Descriptive Statistics**

|  | **N** | **M** | **SD** | **Min** | **Max** |
| --- | --- | --- | --- | --- | --- |
| Planning Horizon | 11,478 | 2.98 | 1.22 | 1.00 | 5.00 |
| Age | 11,478 | 55.12 | 5.65 | 23.00 | 83.00 |
| Female | 11,478 | 0.55 | 0.50 | 0.00 | 1.00 |
| Race: Non-White | 11,478 | 0.20 | 0.40 | 0.00 | 1.00 |
| Education | 11,478 | 0.37 | 0.48 | 0.00 | 1.00 |
| Subjective Mortality Risk | 11,478 | 1.67 | 1.92 | 0.00 | 70.00 |
| Annual Income ($) | 11,478 | 48,127.01 | 50,978.79 | 0.00 | 1300000.00 |
| Total Wealth ($) | 11,478 | 193708.80 | 427517.60 | 0.00 | 8961482.00 |
| Total Debt ($) | 11,478 | 3,234.66 | 19898.76 | 0.00 | 900000.00 |
