# Supplementary material for: A lack of financial planning predicts increased mortality risk: Evidence from cohort studies in the United Kingdom and United States
Source: PLoS One. 2023 Sep 27;18(9):e0290506. doi: 10.1371/journal.pone.0290506 (PMC10529586; doi:10.1371/journal.pone.0290506)
Supplement: S2 Table — (DOCX) [file pone.0290506.s002.docx]

**S2 Table. ELSA Descriptive Statistics**

|  | N | M | SD | Min | Max |
| --- | --- | --- | --- | --- | --- |
| Planning Horizon | 11298 | 3.09 | 1.80 | 0.00 | 6.00 |
| Age | 11298 | 63.96 | 10.92 | 20.00 | 99.00 |
| Female | 11298 | 0.56 | 0.50 | 0.00 | 1.00 |
| Race: Non-White | 11298 | 0.03 | 0.16 | 0.00 | 1.00 |
| Education | 11298 | 0.23 | 0.42 | 0.00 | 1.00 |
| Subjective Mortality Risk | 11298 | 3.46 | 6.02 | 0.00 | 100.00 |
| Annual Income (£) | 11298 | 18,687.78 | 13,836.68 | 0.00 | 16907.90 |
| Total Debt (£) | 11298 | 1493.49 | 5472.82 | 0.00 | 160000.00 |
| Total Wealth (£) | 11298 | 203,741.90 | 236,092.70 | 0.00 | 1675949.00 |
